# From leaf to label: A robust automated workflow for stomata detection

**DOI:** 10.1002/ece3.6571

**Published:** 2020-08-19

**Authors:** Sofie Meeus, Jan Van den Bulcke, Francis wyffels

**Affiliations:** ^1^ Meise Botanic Garden Meise Belgium; ^2^ Department of Environment Ghent University Gent Belgium; ^3^ Department of Electronics and Information Systems IDLab‐AIRO Ghent University‐‐imec Zwijnaarde Belgium

**Keywords:** deep learning, deep neural networks, detection, herbarium, optical microscope images, plants, stomata, stomatal density, VGG19

## Abstract

1. Plant leaf stomata are the gatekeepers of the atmosphere–plant interface and are essential building blocks of land surface models as they control transpiration and photosynthesis. Although more stomatal trait data are needed to significantly reduce the error in these model predictions, recording these traits is time‐consuming, and no standardized protocol is currently available. Some attempts were made to automate stomatal detection from photomicrographs; however, these approaches have the disadvantage of using classic image processing or targeting a narrow taxonomic entity which makes these technologies less robust and generalizable to other plant species. We propose an easy‐to‐use and adaptable workflow from leaf to label. A methodology for automatic stomata detection was developed using deep neural networks according to the state of the art and its applicability demonstrated across the phylogeny of the angiosperms.
2. We used a patch‐based approach for training/tuning three different deep learning architectures. For training, we used 431 micrographs taken from leaf prints made according to the nail polish method from herbarium specimens of 19 species. The best‐performing architecture was tested on 595 images of 16 additional species spread across the angiosperm phylogeny.
3. The nail polish method was successfully applied in 78% of the species sampled here. The VGG19 architecture slightly outperformed the basic shallow and deep architectures, with a confidence threshold equal to 0.7 resulting in an optimal trade‐off between precision and recall. Applying this threshold, the VGG19 architecture obtained an average *F*‐score of 0.87, 0.89, and 0.67 on the training, validation, and unseen test set, respectively. The average accuracy was very high (94%) for computed stomatal counts on unseen images of species used for training.
4. The leaf‐to‐label pipeline is an easy‐to‐use workflow for researchers of different areas of expertise interested in detecting stomata more efficiently. The described methodology was based on multiple species and well‐established methods so that it can serve as a reference for future work.

## Graphical abstract

Our paper proposes a newly developed methodology for automatic stomata detection using deep neural networks according to the state of the art and demonstrates its applicability across the phylogeny of the angiosperms.

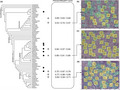

## 1 INTRODUCTION

The study of ecosystem functioning requires a thorough understanding of the physiological processes of organisms occurring at the individual level. Organisms can be defined in terms of their functional traits, which are the phenotypic characteristics that are related to the fitness and performance of an organism. The spatial distribution of these functional traits in combination with environmental conditions constitutes the global diversity in ecosystem functioning and is therefore essential building blocks of land surface models (LSM). LSM are essential for estimating transpiration and photosynthesis from vegetated surfaces (Jefferson, Maxwell, & Constantine, 2017), the dominant component of global land evapotranspiration, and are a key component in models for operational predictions of the near‐climate (Kushnir et al., 2019; Bertolino, Caine, & Gray, 2019). Transpiration in an ecosystem, in essence, occurs at the individual leaf surface where stomata function as “gates” between deep‐soil water reservoirs and the atmosphere. Leaf stomata are microscopic pores surrounded by two guard cells ranging from approximately 10 to 100 µm in length. They control the balance between water loss and CO_2_ uptake by the leaves and therefore have an important effect on the global carbon and hydrologic cycle (Berry, Beerling, & Franks, 2010; Steinthorsdottir, Woodward, Surlyk, & McElwain, 2012; Wang et al., 2015). Moreover, as stomatal traits show a clear response to environmental parameters such as climate (Liu, He, et al., 2018) and atmospheric carbon dioxide concentrations (e.g., Woodward, 1987; Tanaka, Sugano, Shimada, & Hara‐Nishimura, 2013), they are key proxies of environmental change (Hetherington & Woodward, 2003). Stomatal conductance (gs), defined as the uptake rate of carbon dioxide or water vapor loss through the stomata of a leaf, is an elemental parameter in the LSM linking plant water use and carbon uptake (Kala et al., 2016) and is constrained by and derived from the size and density of the leaf stomata (Drake, Froend, & Franks, 2013). It is well known that (maximum and minimum) stomatal conductance, as well as stomatal size, density, and rate of response, varies widely across plant species. Recent efforts have mapped stomatal behavior globally (a.o. Lin et al., 2015), yet more detail is needed as including more interspecific trait variation in climate models could significantly reduce the error in model predictions (Butler et al., 2017; Wolz, Wertin, Abordo, Wang, & Leakey, 2017). To be useful in global‐scale mapping, functional traits should be relatively easy and inexpensive to measure in a large number of taxa using a standardized protocol (Cornelissen et al., 2003; Dawson et al., 2019; Moretti et al., 2017; Pérez‐Harguindeguy et al., 2013). Recording stomatal traits is widely considered to be labor‐intensive and time‐consuming and, to this day, mostly performed manually (e.g., counting stomata through the microscope) and therefore not replicable. The aspects of the methodologies currently used that add to the cost and intensity of the labor are the (a) preparation of the leaves to be viewed with a microscope, (b) the number of replicates to account for the intraindividual variation in stomatal traits, and (c) the measurements, either counts or size measurements, themselves. Few methods to automate the detection of and measurement on stomata have been reported in the literature, and in most cases, they consist of conventional image processing using algorithms that have to be tweaked to the specific task at hand. Scarlett, Tang, Petrie, and Whitty (2016) for instance, apply maximum stable external regions to detect potential ellipses of stomata on microscope images of vine leaves while da Silva Oliveira et al. (2014) use Gaussian filtering and a series of morphological operations to detect stomata on optical microscope imagery of five different plant species. Duarte et al. (2017) use wavelet spot detection in tandem with standard image processing tools to segment stomata on one plant species, and Higaki et al. (2014) combine a genetic algorithm and self–organizing maps, coined clustering‐aided rapid training agent, for the detection of stomata on fluorescently labeled cell contour images of the leaf epidermis of *Arabidopsis* leaves. A series of other papers relies on classifiers for detecting of stomata. Vialet‐Chabrand and Brendel (2014) report on the use of a cascade classifier for rapid assessment of the density and distribution of stomata on the leaves of two oak species. By training a Haar feature‐based classifier with exemplary stomata, they can be detected with high accuracy on *SEM* microphotographs. Jayakody, Liu, Whitty, and Petrie (2017) use a cascade object detection learning algorithm to correctly identify multiple stomata on rather large microscopic images of grapevine leaves, but also apply a combination of image processing techniques to estimate the pore dimensions of the stomata that were detected with the cascade object detector. Typically, the applied classic image processing techniques are based on handcrafted features for the detection and segmentation of the desired stomata. While these techniques perform well on one specific plant species, they do not generalize to other species.

An answer to the limitations of classical image processing techniques came from the field of neural networks with the introduction of deep learning. In a significant breakthrough, Krizhevsky, Sutskever, and Hinton (2012) showed that deep learning was capable of achieving record‐breaking results for object recognition. Deep learning allows computational models that are composed of multiple processing layers to learn representations from raw data with multiple levels of abstraction (LeCun et al., 2015; Najafabadi et al., 2015). Since then, deep learning was quickly adopted by the vision community, which led to state‐of‐the‐art results for the prediction of galaxy pictures (Dieleman, Willett, & Dambre, 2015), face recognition (Parkhi, Vedaldi, & Zisserman, 2015), or the detection of anatomical structures (Shen, Wu, & Suk, 2017; Hoo‐Chang et al., 2016). Its application is now being explored in different fields of biology including plant phenotyping (e.g., Pound et al., 2017) and taxonomy (e.g., Wäldchen and Mäder, 2018). The very recently published work of Fetter, Eberhardt, Barclay, Wing, and Keller (2019), covering 82 angiosperm families, is a good example of the potential of using DL for stomata counting. LeCun et al. (2015) state that all of these successes in deep learning can be explained by the increase in computing power via GPUs, the ease with which data can be collected and various improvements for neural network techniques. Moreover, with the advent of deep learning toolboxes such as Keras (Chollet, 2015), deep learning also became accessible for noncomputer scientists. Although deep learning can outperform other machine‐learning algorithms, training data are needed. Despite their important function, no standardized methodology has yet been described to measure stomatal traits such as stomatal density and size. The handbook of protocols for the measurement of plant functional traits (Cornelissen et al., 2003) highlights the importance of stomata as hard functional traits; however, it does not include any advice standardized way on how to prepare, image and count them, while there is a clear need in the framework of global efforts on the one hand (Lin et al., 2015), and to feed our deep learning networks on the other hand.

Finally, the recent paper by (Christin, Hervet, & Lecomte, 2019) highlights the importance of guidelines and recommendations to help ecologists get started with deep learning. Although deep learning has proven its potential in a lot of disciplines, developing a deep learning solution is not yet a trivial task. They strongly advocate a stronger interaction between computer scientists and ecologists.

However, given the diversity of stomatal shapes and sizes among plants (there are more than 400 angiosperm families only; Haston, Richardson, Stevens, Chase, & Harris, 2009), the variation in techniques for making stomatal impressions (Gitz & Baker, 2009) and the different kinds of imaging techniques available (optical, fluorescence microscopy and *SEM*), there is a clear need for researchers to be able to understand the entire process "from leaf to label" and to tweak this to their own needs. Here, we describe a step‐by‐step guide of a pipeline of actions we have developed from leaf preparation to microscope imaging that is easy, inexpensive, and acquires enough image quality to train and use the DL network. The objective of this paper is therefore twofold. (a) We provide a methodological protocol aimed at standardizing sample preparation as well as imaging of stomata. The rationale is to facilitate comparability and usability across studies for revealing patterns and mechanisms by increasing the reliability and predictive power of stomatal counts. More specifically, we outline an accessible methodology to obtain stomatal counts “from leaf to label” that can be applied beyond a laboratory setting and is also suitable for educational purposes. (b) We present a tutorial‐styled detailed and replicable methodology for automatic stomata detection with deep neural networks and show its applicability of deep learning across the phylogeny of the angiosperms. Our aim is to motivate researchers from the ecology and evolution community to consider deep learning techniques for the automation of their workflows.

## 2 MATERIALS AND METHODS

### 2.1 Dataset generation

#### 2.1.1 Specimens and species

We used mounted specimens from the African herbarium collection of Meise Botanic Garden which contains approximately 500,000 herbarium specimens from Burundi, Rwanda, and Democratic Republic of the Congo, representing more than 80% of the existing collections from these countries (Stoffelen P., pers. comm., 2019). Five fully developed leaves per specimen were carefully detached and remounted afterward. The species for algorithm training were selected in the context of studying the effects of global change on the central African forest vegetation (Bauters et al., 2020). The specimens used here mainly came from common tropical (timber) tree species such as *Cola griseiflora*, *Mammea africana*, and *Erythrophleum suaveolens* which are well represented in the collection and were recurrently collected throughout the last century (1902–2013; for complete species list, see Table S1). These herbarium specimens were collected at the Yangambi Biosphere Reserve, situated within the Congo River Basin west of the City Kisangani in the Democratic Republic of the Congo.

#### 2.1.2 Leaf prints

Surfaces of plant leaves are very variable in texture, so depending on the species of study one may want to try different approaches for visualization of the stomata. Many methods for looking at stomata exist and can be categorized into two classes: the use of fresh leaf material or imprints. Important criteria for choosing a method are as follows: toxicity, availability of a laboratorium space, negative versus positive image, effect on stomatal movements, preferred or available microscopic visualization technique (e.g., light microscopy, scanning electron microscopy), slide preservation, damage to the leaf tissue, and ease of use. For this study, we opted for the traditional nail polish method because we needed a nondestructive technique for generating leaf prints as we are dealing with valuable historical dried plant material. However, for some thin‐leaved species, this method was not suited. Also for our purposes, the preserving and archiving of impressions were of minor importance compared to the ease of use, as was the need for a positive replica or the effect of the technique on stomatal movements. For a comprehensive comparison of the quality of different techniques for creating stomatal impressions in combination with brightfield microscopy, we refer to Gitz and Baker (2009). Epidermal leaf impressions were made from the abaxial side of the leaves in the middle of the leaf, between the midvein and edge. This region of the leaf has been shown to contain guard cell lengths and stomatal densities comparable to the means of the entire leaf (Beaulieu, Leitch, Patel, Pendharkar, & Knight, 2008, and references therein). Transparent nail polish (Bourjois Crystal ball) was used to make the impressions which, once dried, were mounted pointing upward with double‐sided tape (Scotch™) on a microscope slide.

#### 2.1.3 Image acquisition

Three photomicrographs of 1,600 × 1,200 pixels were taken per leaf print (dimensions = 344 × 258 µm; area view field = 0.09 mm^2^) using a digital microscope (VH‐5000 Ver 1.5.1.1, KEYENCE CORPORATION) with full coaxial lightning and default factory settings for shutter speed at × 1,000 lens magnification (VH‐Z250R). A single photomicrograph was created by stacking of several digital images taken at different focal planes to increase the depth of the resulting image. All stomata that fell entirely within the view field were counted and converted to stomata per square millimeter to obtain stomatal density.

### 2.2 Model development

#### 2.2.1 Deep learning approach

**Figure 1:**
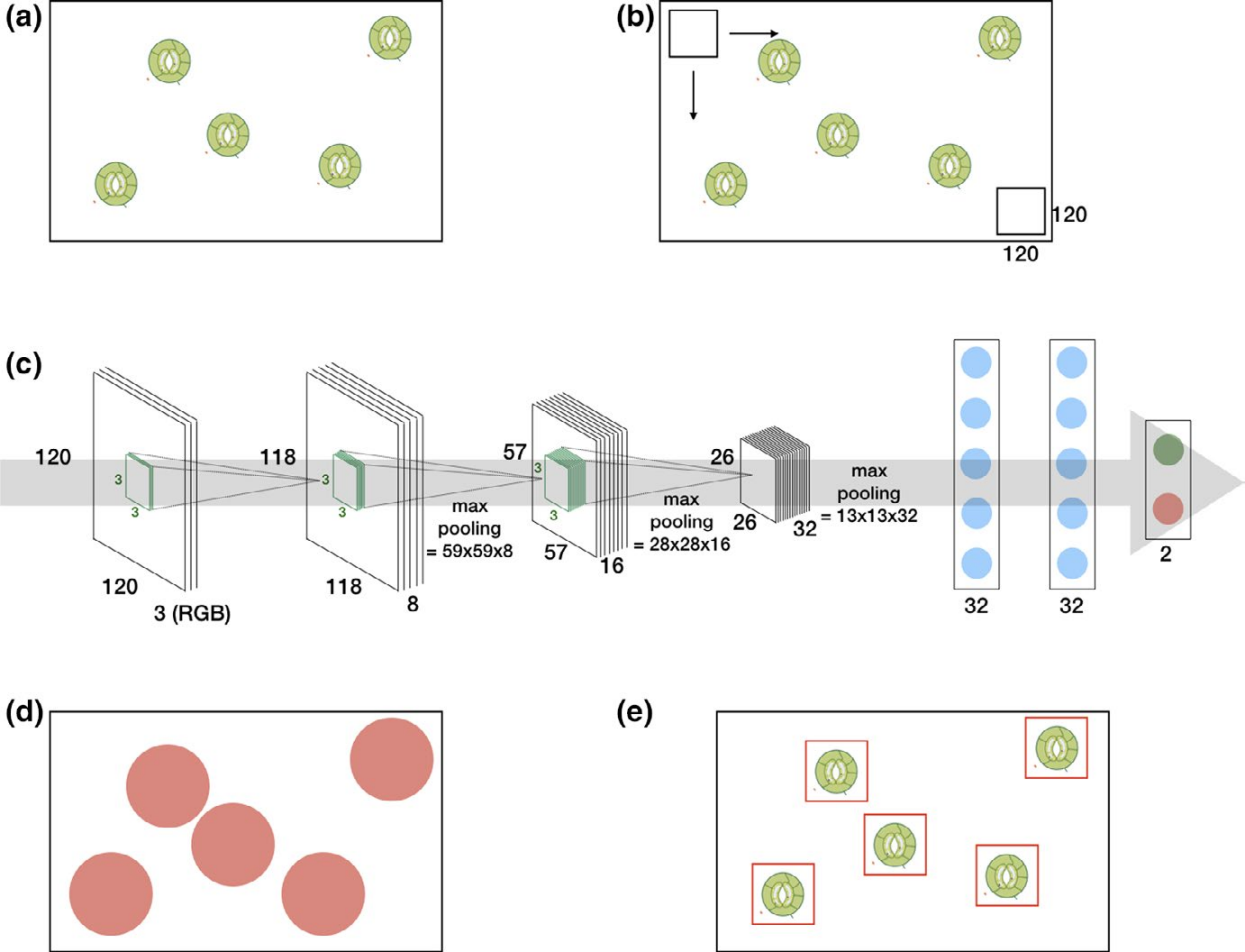
From leaf to label: a simple deep learning approach for automatic stomata detection. A photomicrograph (a) is divided into small overlapping patches (b) by using a sliding window approach. The deep learning architecture (c) is trained to label these patches. Positively labeled patches of a photomicrograph (d) are clustered which results in the detection (e)

A basic deep learning architecture is depicted in Figure 1c. It consists, from left to right, of an input layer, multiple stacked convolutional and pooling layers, a fully connected feedforward neural network, and an output layer. By alternating convolutional and pooling layers, the (raw) input (e.g., a RGB image) is progressively transformed into more abstract representations. Therefore, the convolutional layers convolve the input feature maps with a set of learnable filters (i.e., nonlinear transformations) to produce a stack of output feature maps (Zeiler & Fergus, 2014). The pooling layers are used to reduce the dimensionality of the feature maps by computing some aggregation function (typically the maximum or the mean) across small local regions of the input (Boureau et al., 2010). This results in a hierarchical set of features where higher‐level (more abstract) features are defined in terms of lower‐level (less abstract) features (Najafabadi et al., 2015).

The resulting feature maps are then concatenated and fed into a stack of fully connected neural layers to map these features onto the desired output.

Deep neural networks come with a lot of trainable parameters: an order of magnitude of a hundred million parameters is not uncommon. In order to properly adjust the weights, gradient descent in combination with the backpropagation procedure can be used (LeCun et al., 2015). By applying the chain rule on the stacked layers on both the convolutional and fully connected layers, the gradient of the objective with respect to the input can be computed. The backpropagation equation can be applied repeatedly to propagate gradients through all modules, starting from the output at the top (where the network produces its prediction) all the way to the bottom (where the external input is fed) (LeCun et al., 2015). Today, adapted versions of the gradient descent optimization algorithm are used (see Ruder, 2016, for an overview). A particular popular optimizer is Adam (Kingma & Ba, 2014), an adaptive learning‐rate method, with bias correction and momentum.

Because of their proven capabilities and state‐of‐the‐art results in many domains, deep neural networks are popular. However, due to their huge amount of trainable parameters, overfitting to data remains a major challenge. A toolbox of techniques to avoid overfitting exists, including the reduction of the model complexity by reducing the number of hidden layers or units, layer‐wise pretraining and fine‐tuning (Bengio, Lamblin, Popovici, & Larochelle, 2007), dropout (Srivastava, Hinton, Krizhevsky, Sutskever, & Salakhutdinov, 2014), and data augmentation (Simard, Steinkraus, & Platt, 2003).

#### 2.2.2 Detection of stomata with deep learning

In this work, we assessed the performance of deep learning for the detection of stomata. While this task can be broadened to a generic object detection task for which multiple efficient methodologies were proposed (see Liu, Ouyang, et al., 2018, for an extensive review), we focus on a simple methodology across multiple species which models the stomata detection task as classification task within a fixed window. This baseline approach is illustrated in Figure 1.

**Figure 2:**
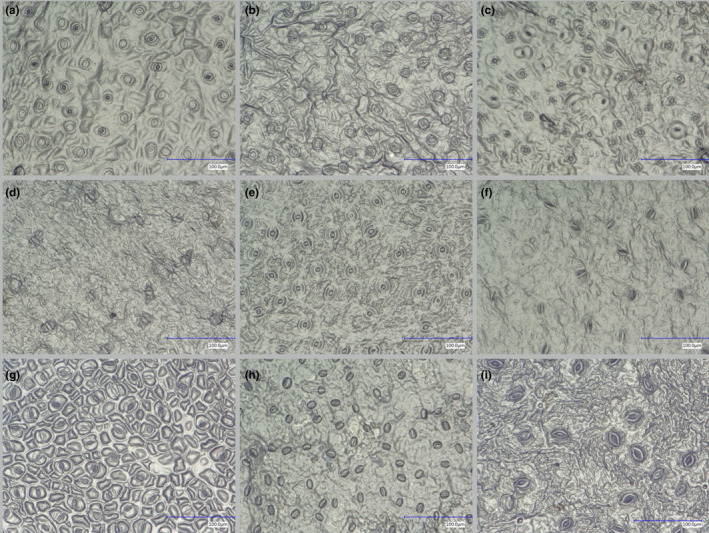
Stomata microscope images of herbarium specimens of nine representative species of the training set used to train the classification algorithm: *Cola griseiflora* (a), *Carapa procera* (b), *Celtis mildbraedii* (c), *Garcinia punctata* (d), *Mammea africana* (e), *Petersianthus macrocarpus* (f), *Prioria balsamifera* (g), *Erythrophleum suaveolens* (h), *Trichilia gigliana* (i)

For generating the training set, we used herbarium specimens of 19 common tropical tree species belonging to 12 flowering plant families and eight orders (Figure 2, Table S1). The choice of training set was made in function of a running research project (COBECORE) to investigate the change in stomatal density and function over time in Central African tropical rainforest (Bauters et al., 2020). A total of 431 micrographs were used for training, 1–53 training images per species, 3–115 per family, and 14–126 per order.

**Figure 3:**
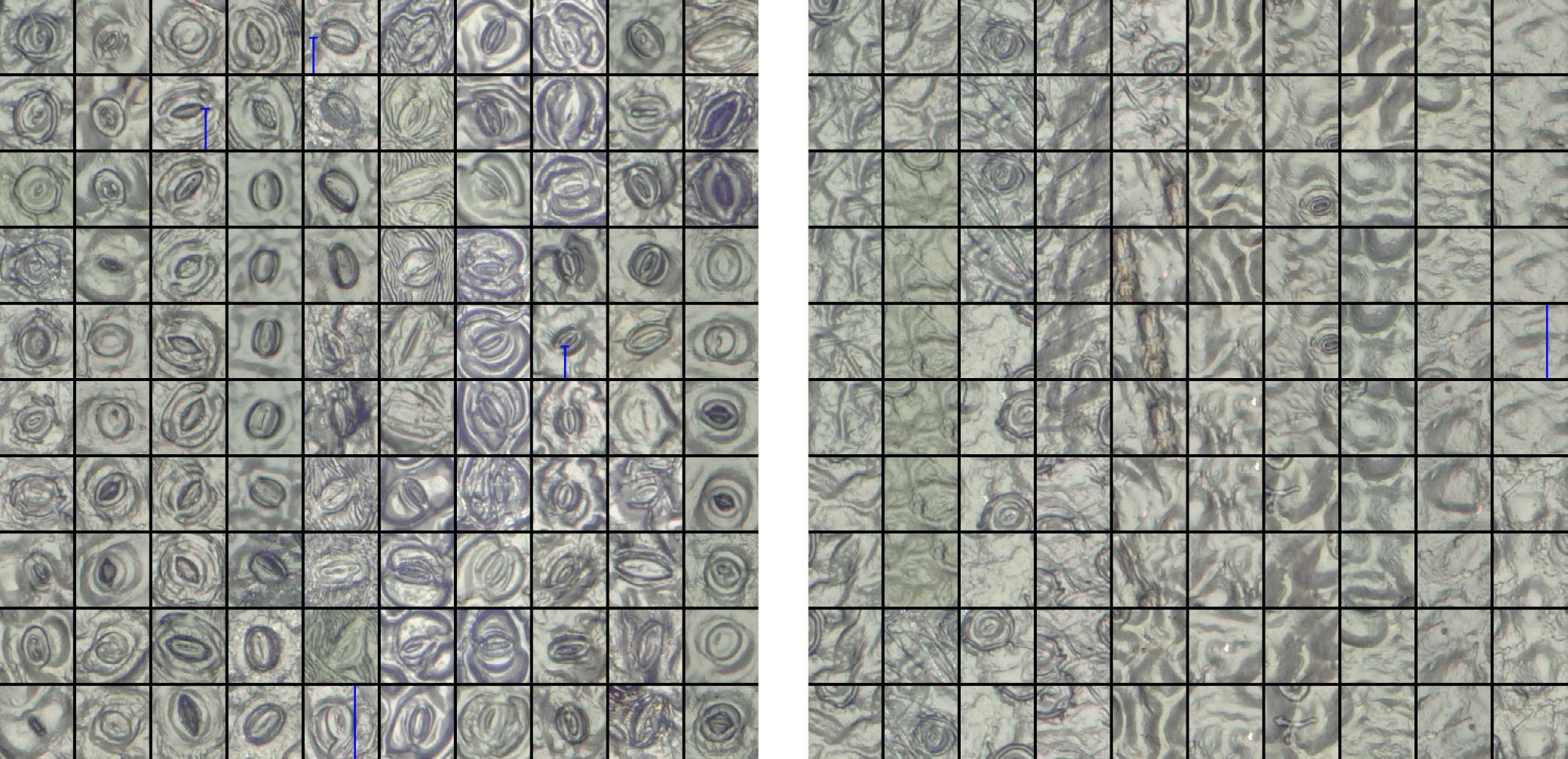
Patches with positive (left) and negative (right) examples of stomata. Stomata that are not fully visible were labeled negative

In order to detect the stomata in a picture, we applied a simple patch‐based method (Cruz‐Roa et al., 2014; Hou et al., 2016). Therefore, we divided each picture in multiple overlapping patches of size 120‐by‐120 pixels. This patch size is based on the average stomatal size observed in the training set. The patches were labeled as being positive or negative by an expert (Figure 3). Note the variability of the stomata in the training set as well the variability of the negative patches due to the occurrence of different artifacts in the data. In total, we extracted more than 12 thousand positive labeled patches and 72 thousand negative patches from the training set. Due to the apparent larger variability in the negative patches, more negative patches than positive patches were included.

The obtained patches were then used to train three different deep learning models: two basic architectures with three convolutional layers followed by two dense layers and an output layer (Figure 1c) with, respectively, 180, 242, and 23,297,090 trainable parameters. Therefore, we varied the depth of the convolutional layers from 8–16–32 (basic shallow) and 32–64–128 (basic deep), and the size of the dense layers: 2 × 32 neurons (basic shallow) and 2 × 1,024 neurons (basic deep). One VGG19 (Simonyan & Zisserman, 2014) architecture with 47,297,602 parameters of which 27,273,218 were trained (i.e., the parameters from the fully connected layers) by fine‐tuning them on our training set and 20,024,384 parameters (i.e., the parameters from the convolutional layers) obtained through pretraining on ImageNet (Deng et al., 2009). These parameters were optimized by using the Adam (Kingma & Ba, 2014) learning rule for which both the batch size and learning rate were tuned. Dropout and data augmentation, by random rotations, horizontal and vertical flips of the patches, were applied to avoid overfitting. Table 1 summarizes all the training parameters of the deep learning architecture. Our deep learning models were trained (or fine‐tuned in the case of VGG19) over 200 epochs (50 epochs for VGG19) to output two numbers between 0.0 and 1.0 indicating the absence or presence of a stoma. Intuitively, the output is either [0.0, 1.0] or [1.0, 0.0] depending on whether the patch contains the whole stoma or not. In reality, however, the network will output any number between 0.0 and 1.0 depending on the model confidence. Consequently, one has to tune a threshold with a validation set which, in this case, consisted of three plant species belonging to the order of Sapindales (*Lannea acida, Lannea welwitschii,* and *Lannea schweinfurthii*) and are relatively closely related to the species from the training set (see Table S1). Lastly, all positively labeled patches are clustered by using mean shift clustering (Comaniciu & Meer, 2002). This technique groups neighboring (or even overlapping) positively labeled patches from which the resulting stoma coordinates are derived. All software was implemented in Python 3.6. Keras (Chollet, 2015) and Tensorflow (Abadi et al., 2016) were used to implement the deep learning models. Training and testing were performed on a Linux (Ubuntu 18.04) workstation with an i7‐5930k CPU, 64 GB RAM, and a Nvidia™ Titan Xp GPU.

**Table 1:** Summary of the training parameters

Parameter	Basic shallow	Basic deep	VGG19
Number of parameters	180,242	23,297,090	47,297,602
Number of trainable parameters	180,242	23,297,090	27,273,218
Optimizer	Adam	Adam	Adam
Parameters optimizer	*α* = 5e−4	*α* = 5e−5	*α* = 5e−6
	*β*_1_ = 0.9	*β*_1_ = 0.9	*β*_1_ = 0.9
	*β*_2_ = 0.999	*β*_2_ = 0.999	*β*_2_ = 0.999
Batch size	32	64	128
Training epochs	200	200	50

To evaluate the performance of the model, we calculated the information retrieval (IR) standard measures, precision $\left(=\frac{\text{TP}}{\text{TP}+\text{FP}}\right)$ and recall $\left(=\frac{\text{TP}}{\text{TP}+\text{FN}}\right)$. Precision decreases with the number of false positives (FP) and recall with the number of false negatives (FN). The *F*‐score is the harmonic mean of precision and recall with a high *F*‐score, meaning low false positives and low false negatives. Precision indices were calculated for all annotated images used for training (denoted "training set" in Table S1), on 70 unseen images from a subset of the training set (unseen within the scope of training) and on 595 images from species not included in the training set (unseen beyond the scope of training), a range of 16 species from seven genera chosen from more and less related angiosperm orders as the samples used for training. The latter set was included to assess the performance of the model on other angiosperm species and to evaluate how well the model generalizes to these other species. We expected the deep learning model to perform better on species from the same angiosperm order as the training species as related species are expected to resemble each other more in stomatal shape and size (Zhang et al., 2012). As stomatal shape can vary in relation to climate even between species within a genus (e.g., Pautov et al., 2017; Yukawa, Ando, Karasawa, & Hashimoto, 1992), we sampled for this dataset three species within each genus with one species from tropical rainforest, one from tropical moist deciduous forest and one from tropical shrubland and desert (but only two climate regions for the Asparagaceae and one for Orchidaceae) to average precision measures and be able to compare genera by controlling for provenance. Precision indices for the training set were calculated to assess the performance in function of the number of stomata used per species for training and to compare performance to the "unseen beyond the scope of training" set.

The output of the developed model for stomatal detection consists of the coordinates of the detected stomata. To calculate stomatal densities for scientific research questions, all stomata per image are counted and converted to the number of stomata per square millimeter. The accuracy (%) was calculated for 70 unseen images of species used in the training set (unseen within the scope of training) for which we compared manual and computed stomatal counts. Accuracy is defined as the ratio of the number of correctly classified items to the total number of items (Michie et al., 1994). Goodness of fit was determined by calculation of the coefficient of determination (*R* ^2^) from a linear regression between computed and manual counts.

## 3 RESULTS

### 3.1 Efficacy of the nail polish method

A total of 49 species were sampled from the African herbarium of Meise Botanic Garden (Table S1). The nail polish method was successfully applied in 78% of the species sampled. Generating impressions failed in 16% of the species due to hairy or velvety leaf surfaces. In 7% of the species for which we managed to get leaf prints, we were unable to detect the stomata visually.

### 3.2 Model selection and evaluation

**Figure 4:**
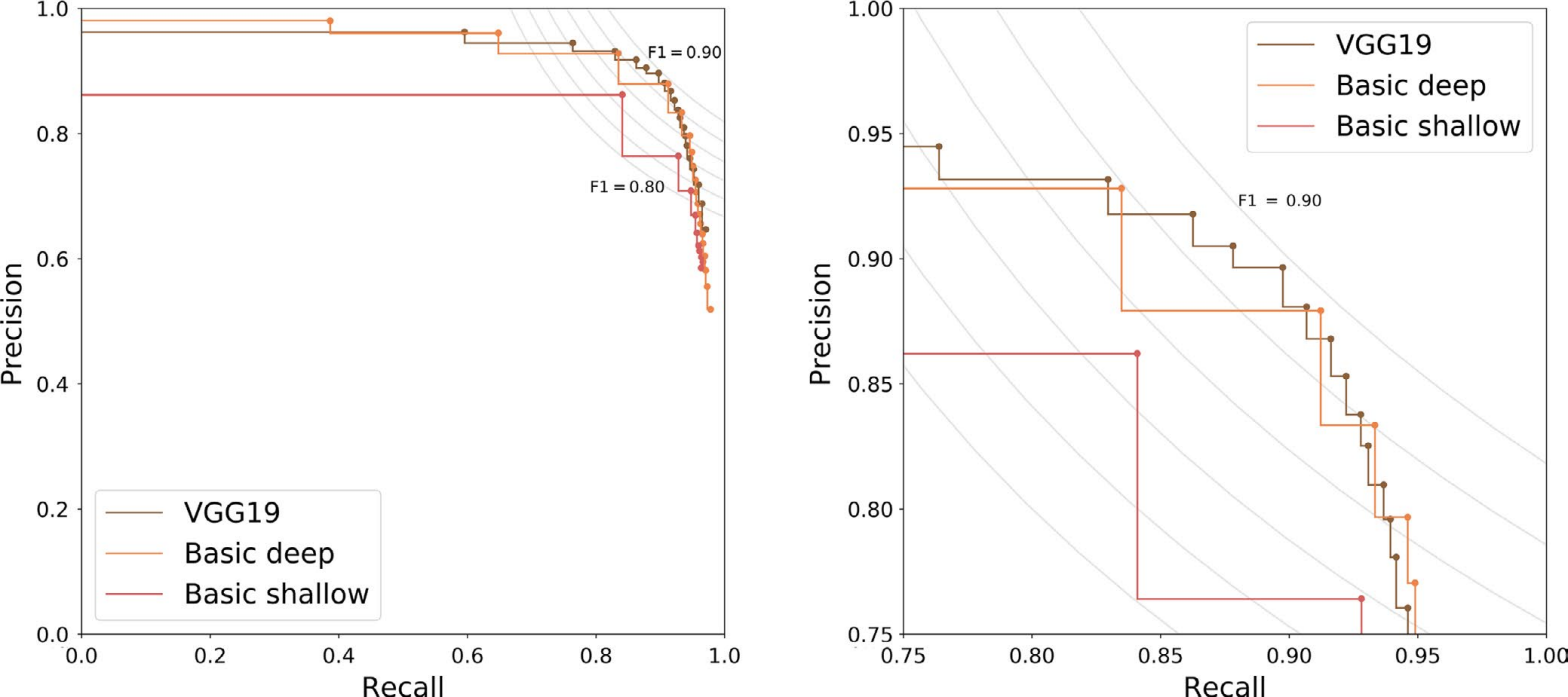
Precision–recall curve for the detection of three species unseen during training for three different deep learning architectures (zoom on the right). The curve gives us insight on how to choose the decision threshold which ranged from 0.05 to 0.95 in steps of 0.05. To guide this decision process, the *F* iso‐curves are shown as well

The accuracy of all three architectures on an unseen dataset (i.e., the validation set) is depicted in Figure 4, illustrating the precision and recall for varying thresholds (0.05–0.95), as well as the *F*‐score, which is an indication of the overall performance. One can observe that there is a trade‐off between precision and recall. For example, one can choose to obtain maximal precision with very low recall or vice versa. From Figure 4, it is clear that with increasing trainable parameters, the performance of the architecture increases, although the VGG19 architecture only slightly outperforms the basic architectures. Furthermore, from Figure 4, one can observe that the VGG19 architecture is less sensitive to the choice of the threshold in comparison with the basic architecture. Moreover, VGG19 can be seen as a standard textbook approach, while the basic architecture was hand‐tuned. For all these reasons, we will continue our analysis and discussion with the VGG19 architecture. However, we want to point out that our choice is not the computationally most efficient. With less parameters, the basic architectures are less computationally demanding than VGG19. We refer to the work of Bianco, Cadene, Celona, and Napoletano (2018) for a benchmark study of deep learning architectures.

**Figure 5:**
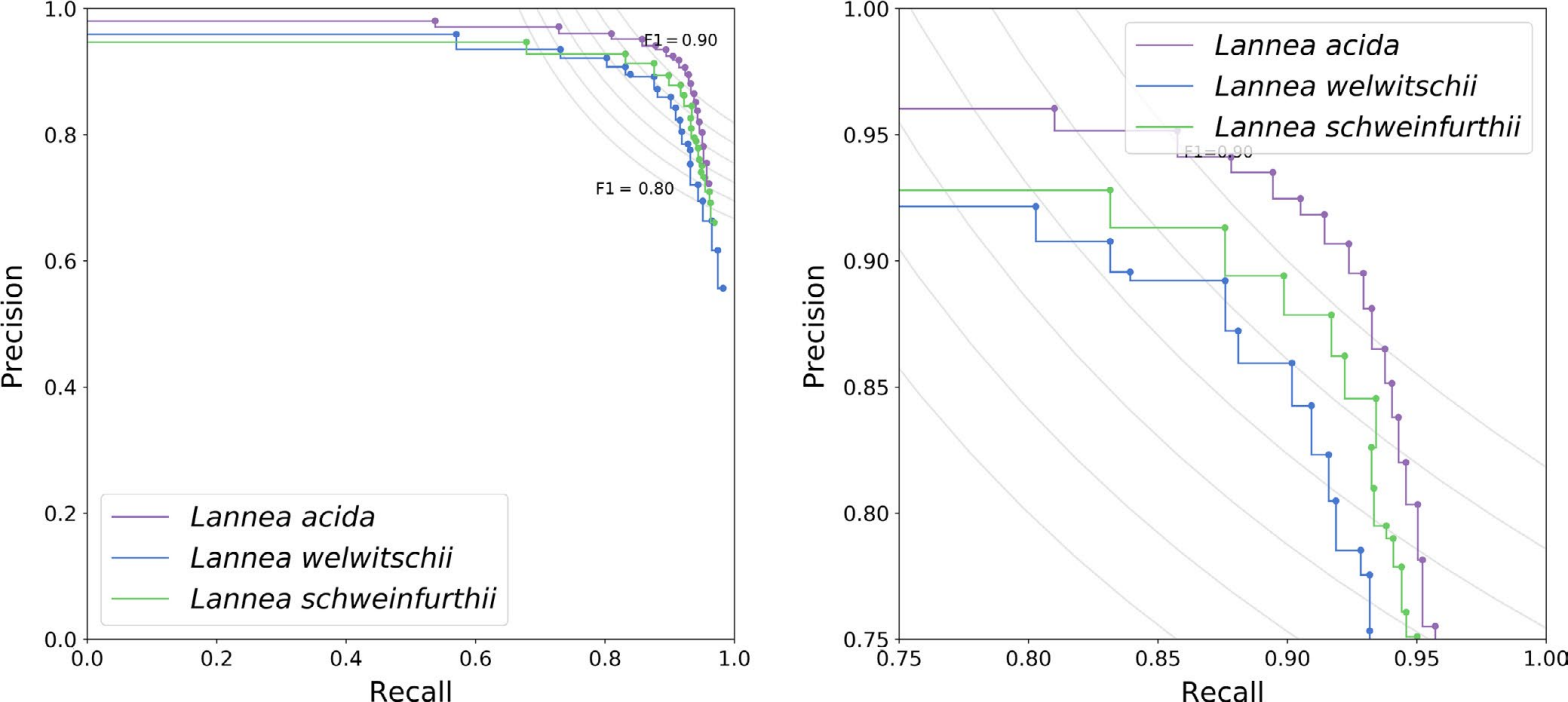
Precision–recall curve for the fine‐tuned VGG19 architecture on the three different species (zoom on the right)

**Figure 6:**
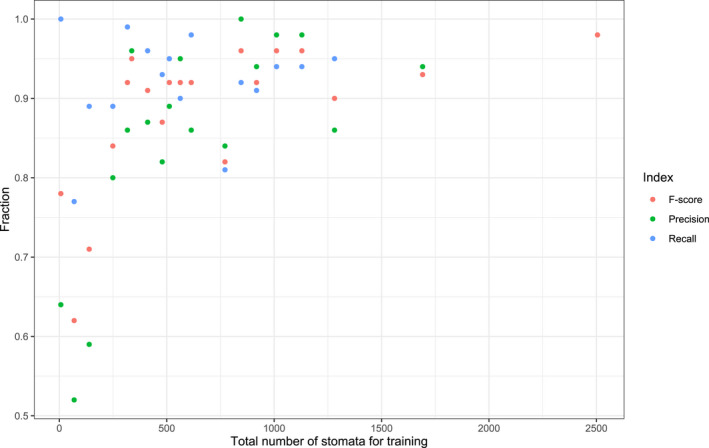
Precision, recall, and *F*‐score indices in function of the number of stomata used for each of the 19 species for training

Figure 5 shows that there are slight variations of the performance on the validation set. For the VGG19 architecture, a threshold equal to 0.7 is a good trade‐off between precision and recall and will result in an average *F*‐score of 0.89. This is close to an average *F*‐score of 0.87 for the plant species of the training set. For species for which 250 stomata or more were used for training, precision, recall, and *F*‐score values of 0.8 and higher were obtained (Figure 6).

### 3.3 Accuracy

**Figure 7:**
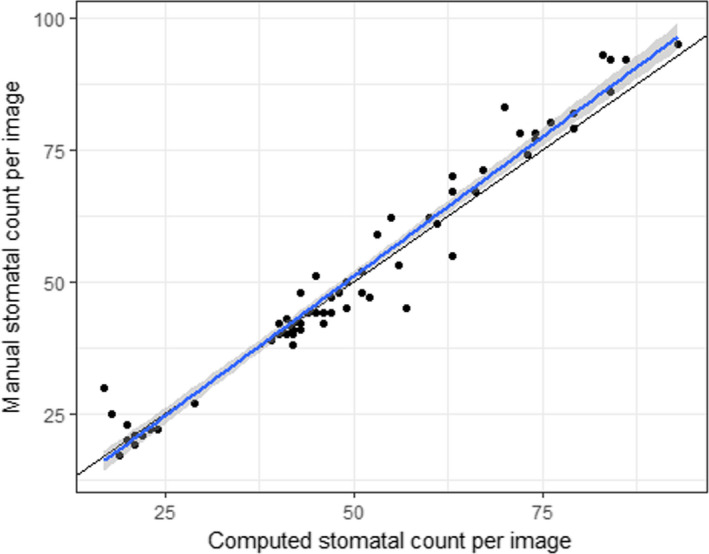
Accuracy of the computed stomatal counts per image (*n* = 70) from seven species included in the training set. The blue line with gray 95% CIs is the regression line with slope 1.056 and intercept −1.8 (*R* ^2^ = .96, *p* < .001); the black line is the reference line (1:1)

The accuracy was calculated for 70 images of species within the scope of the training set to compare results of stomatal densities between computed and manual counts. Average accuracy was high (94%), and a strong correlation between the computed counts and the manual counts was observed among all the images (Figure 7, *R* ^2^ = .96, *p* < .001). Figure 7 shows the reference line (1:1) with an intercept within the 95% confidence interval (CI) around the intercept (−4.46 to 0.86) of the linear regression and with a slope value of 1 slightly outside the 95% CI of the regression slope (1.01–1.11). For images containing many stomata (>60) stomatal number tends to be underestimated (Figure 7).

### 3.4 Generalization to other species

**Figure 8:**
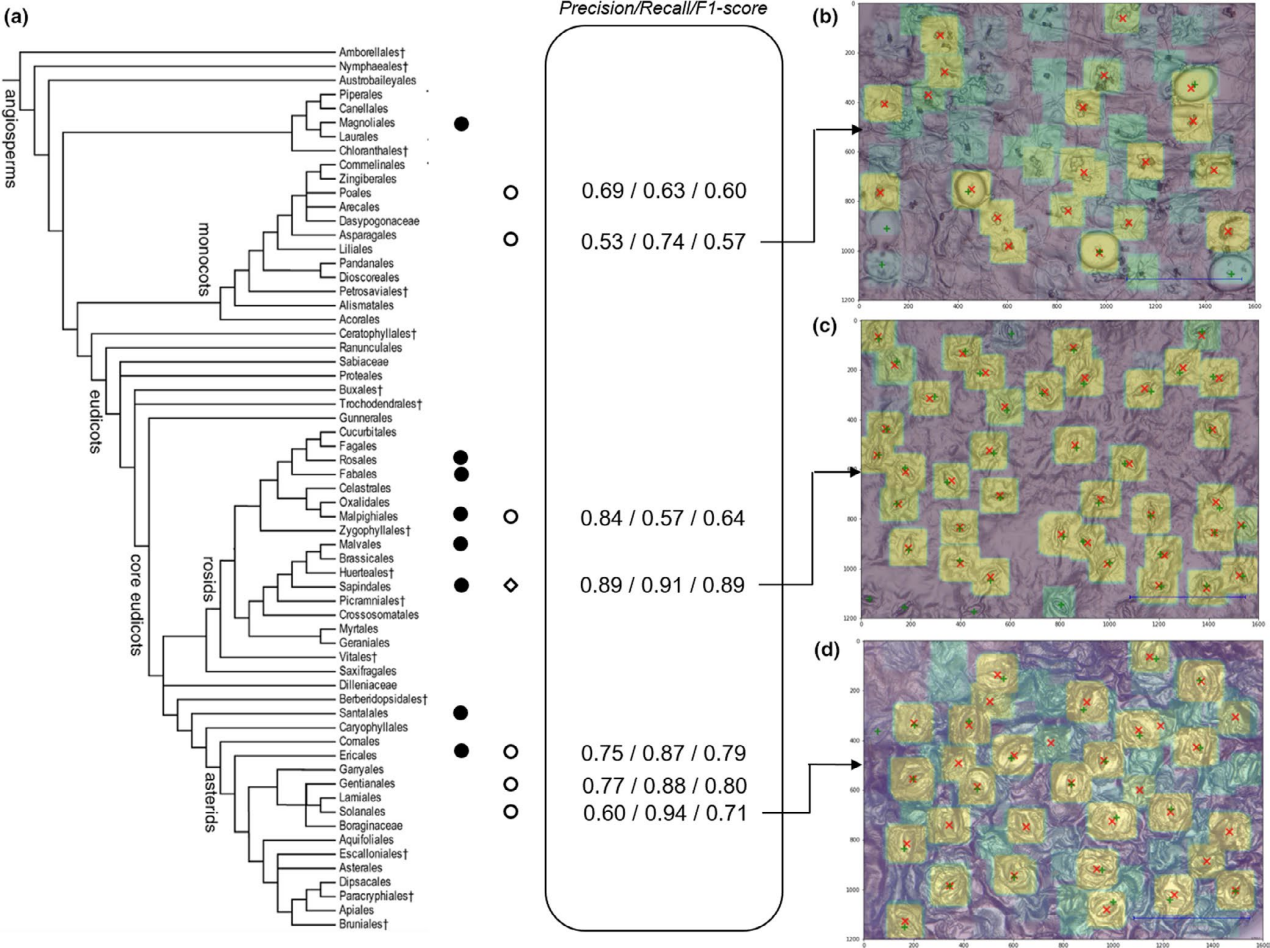
Performance of the network in function of the phylogenetic relatedness of taxa used for training, validation, and testing. (a) Angiosperm phylogeny (modified from APG III, 2009) and diversity in the training set (full circles) and test set (open circles). The open diamond indicates the position within the angiosperm phylogeny of the taxa used for validation (*Lannea* species; see text). The numbers in the central frame denote the performance indices: precision, recall, and *F*‐score. Average precision, recall, and *F*‐score for the training set are 0.84, 0.91, and 0.87, respectively. Images (b, c, and d) visualize the performance of the network on unseen taxa belonging to the test set with (b) *Cyrtorchis chailluana* (Orchidaceae, Asparagales), (c) *Lannea schweinfurthii* (Anacardiaceae, Sapindales), and (d) *Ipomoea eriocarpa* (Convolvulaceae, Solanales). Green crosses denote the actual stomata, red x's the stomata recognized by the network with a confidence of 0.7 or higher. Color gradient from green (low confidence) to yellow (high confidence)

In Figure 8, the overall performance of the VGG19 architecture on the "unseen beyond the scope of training set" (open circles) is shown for a confidence threshold of 0.7. Average precision, recall, and *F*‐score for the training set are 0.84, 0.91, and 0.87, respectively. Performance indices for unseen species within the same angiosperm order as the training set (Malpighiales, Ericales) range between 0.75 and 0.84 for precision, 0.57 and 0.87 for recall, and 0.64 and 0.79 for *F*‐score. Performance indices for unseen species beyond the training set (Poales, Asparagales, Gentianales, Solanales) range between 0.53 and 0.77 for precision, 0.63 and 0.94 for recall, and 0.57 and 0.80 for *F*‐score.

## 4 DISCUSSION

In this work, we developed a leaf‐to‐label workflow that allows detecting stomata on light microscope images from dried plant material such as that of herbarium specimens. Even though mostly used in fresh plant material (e.g., Wu & Zhao, 2017), the nail polish method proves to be a reliable, noninvasive, easy, and inexpensive method that can obtain qualitative leaf impressions from dried leaves on the majority of species (78%). We trained a deep learning architecture for the detection of stomata in focus‐stacked images of high resolution. However, we believe that traditional light microscopy could also be used for imaging given that the entire field of view is in focus. We illustrated that, even with a simple deep learning approach in which we model the object detection problem as a classification problem with a fixed patch size based, a *F*‐score of 0.89 can be reached on unseen taxa on the condition that they are in the phylogenetic scope of the training set. This is in line with the average results (*F*‐score: 0.87) on the training set. The model on average did not perform better on unseen species within the same angiosperm order as the training set (*F*‐score: 0.64–0.79) as compared to its performance on unseen species of other angiosperm orders (*F*‐score: 0.57–0.80). This result seems to indicate that the variation in stomatal structure and shape within flowering plant orders is similar to the variation between them. However, note that this test set includes at most a few species representing an angiosperm order and therefore does not include all variation within genera, families, and orders. The training focused mainly on taxa belonging to the core eudicots and one species of the basal angiosperms *Polyalthia suaveolens* (Magnoliales). The model performed on average better on unseen species from the core eudicots (*F*‐score: 0.77) than on unseen species from the monocots (*F*‐score: 0.59). The difference in stomatal shape between monocots and the dicots is apparent, especially the grasses (Poaceae), represented here by *Chloris* species, are known for their particular dumbbell‐shaped guard cells as compared to kidney‐shaped cells of dicots (Rudall, Chen, & Cullen, 2017; Zeiger, Farquhar, & Cowan, 1987). Also, the orchid species included in the test set, *Cyrtorchis chailluana* has a stomatal shape not easily detected by our model (Figure 8), probably because of its particular circular shape and round opening [*cf*. stoma type II in *Dendrobium* (Yukawa et al., 1992)]. Also note that we did not include species with extremely large stomata typical for, for example, the Liliaceae, as this will decrease the performance of the model to detect the stomata (but see below).

While the model performs relatively well over a broad taxonomy, our approach has room for further improvement. First, the model performance is highly related to the variation (Figure 8), the quantity (Figure 6), and quality of training images. The network presented in this paper is not trained to handle low‐quality images. Therefore, high‐quality images should be aimed to enable the network to perform optimally (see also Fetter et al., 2019). The quality (contrast, blurriness, etc.) of a set of pictures can be quantified using the image histogram and using PyImageQualityRanking software for ranking the images in a set and detecting outliers (Koho, Fazeli, Eriksson, & Hänninen, 2016). Based on this ranking, one can choose to leave out low‐ranked images due to their insufficient quality. If low‐quality images should still be processed, then the network should be trained accordingly.

Second, the performance of our model depends on the number of examples of stomata used during training (Figure 6). In general, if more examples are available of a species, the better the performance is of the model for that species. In this case, even though overall accuracy of stomatal counts was very high (94%) for unseen pictures of seven species (Figure 7), the average accuracy for each of the species individually was highly correlated with the number of images and total number of stomata seen during training (results not shown) as was the case for the information retrieval (IR) standard measures (Figure 6). For example, only 69 stomata or three images for the species *Irvingia grandifolia* were used in training (Table S1) rendering an average accuracy of 64%, that is, a reduction or increase in stomatal density of 36%. Since a 28% reduction in stomatal density in transgenic poplars is enough to cause a 30% drop in transpiration (Bertolino et al., 2019; Wang et al., 2016), we recommend at least 250 stomata for training depending on the level of difference in stomatal density one wants to detect. If small differences in stomatal density within a species are targeted, the general protocol described in this paper can be used. In order to obtain more accurate results, the threshold of the deep learning model (cf. Section 3.2) can be adjusted for each species separately. Furthermore, the accuracy for an individual species can be increased by fine‐tuning the model by training the dense layers of the deep learning model.

Third, our patch‐based approach is constrained by a patch size of 120‐by‐120 pixels which correspond to a window of 25 by 25 µm using the microscope settings as described above. Angiosperms on average have a stomatal length or guard cell length of 31 µm (cf. Beaulieu et al., 2008; Hodgson et al., 2010; Figure S1). Although this patch size could be successfully applied to the majority of angiosperm species, the patch size limits both the aspect ratio and the scale of the input image. The simplest solution is to adjust magnification during data collection, by increasing the magnification when stomata are too small to be detected and decreasing the magnification when stomata extend beyond a patch size of 120‐by‐120 pixels. In our model, stomata between 60 and 120 pixels are best detected by the model. Another more elegant way of handling this problem is by including some region of interest pooling layer as discussed by Dai, Li, He, and Sun (2016) and He, Zhang, Ren, and Sun (2014), which would allow moving from the patch‐based method to detect all stomata in a spatial hierarchical way. This object detection pipeline can be improved further with Fast R‐CNN (Girshick, 2015) and Faster R‐CNN (Ren, He, Girshick, & Sun, 2015) which combine the idea of using a spatial hierarchical pooling with region‐based convolutions into an end‐to‐end trainable deep learning model. Furthermore, if processing speed is an issue, one can opt for a single shot multibox detector approach (SSD). SSD discretizes the output space of bounding boxes into a set of default boxes over different aspect ratios and scales per feature map location (Liu et al., 2016). At prediction time, the network generates scores for the presence of each object category in each default box and produces adjustments to the box to better match the object shape.

Fourth, with the current advances in deep learning, the object detection pipeline can be improved further by using novel convolutional neural network architectures such as Xception (Chollet, 2017) or ResNeXt (Xie, Girshick, Dollár, Tu, & He, 2017) as a backbone for feature extraction. See Bianco et al. (2018) for an in‐depth analysis of the majority of the deep neural network architectures that deviate from the idea that simply stacking convolutional layers is sufficient.

To summarize, we illustrated that by using a simple deep learning architecture one can work out a simple leaf‐to‐label workflow that allows detecting stomata on light microscope images from dried plant material such as that of herbarium specimens. Our approach can be optimized depending on the availability of the data as well as by using more recent object detection pipelines. We recommend the survey paper of Liu, Ouyang, et al. (2018) and Huang et al. (2017) for a thorough overview and benchmarking of object detector pipelines.

## 5 CONCLUSIONS

The entire leaf‐to‐label pipeline presented here could be of use in different research areas with the need for stomatal count data of many specimens. It will allow ecologists to focus on the ecological questions rather than on the technical aspects of data analysis and more specifically deep learning, and computer scientists to pave new roads on some of the biological world's most complex units, such as ecosystems (Christin et al., 2019). Large‐scale studies using stomata of fossils to reconstruct a changing environment in deep time (e.g., Franks, Berry, Lombardozzi, & Bonan, 2017; Mcelwain, Beerling, & Woodward, 1999), as well as work on the anthropogenic effect on stomatal density and size in agricultural crops (Zheng et al., 2013), could benefit from such an approach, that is, the use of a general deep learning model that can be tweaked and expanded for the detection of other objects such as epidermal cells. Especially the information locked in the archives of herbaria, the result of century‐long efforts of collecting, has shown to be of great value in several studies, as the digitization of herbaria specimens has the potential to produce data to facilitate the study of the natural world (Goodwin, Harris, Filer, Wood, & Scotland, 2015). The leaf‐to‐image approach described here is easy to perform and given that imaging technology is becoming faster and can be partially automated, the exploration of these sleeping beauties is within reach.

## CONFLICT OF INTEREST

None declared.

## AUTHOR CONTRIBUTIONS

**Sofie Meeus**: conceptualization (equal); data curation (lead); formal analysis (equal); methodology (supporting); project administration (lead); resources (lead); software (supporting); supervision (equal); writing—original draft (equal); writing—review editing (equal). **Jan Van den Bulcke**: conceptualization (equal); formal analysis (supporting); methodology (equal); project administration (supporting); software (supporting); supervision (supporting); writing—original draft (equal); writing—review editing (equal). **Francis wyffels**: conceptualization (equal); data curation (equal); formal analysis (equal); methodology (lead); project administration (supporting); resources (equal); software (lead); supervision (equal); writing—original draft (equal); writing—review editing (equal).

## Supporting information

Supinfo

## Data Availability

The trained model is accessible to use at https://kiks.ilabt.imec.be/ for the upload of individual images. The do‐it‐yourself tutorial for training and detection as well as an example image set can be accessed on GitHub by following this link: http://github.com/fwyffels/LeafToLabel. The example image set can also be downloaded here: http://doi.org/10.5281/zenodo.3902280. All light microscope images used in this study are made freely accessible on Zenodo under the CC‐by license (http://doi.org/10.5281/zenodo.3579227). The herbarium specimens as referred to in Table S1 can be visualized in the virtual herbarium of Meise Botanic Garden following this link: http://www.botanicalcollections.be/specimen/barcode.
