# Supplementary material for: From leaf to label: A robust automated workflow for stomata detection
Source: Ecol Evol. 2020 Aug 19;10(17):9178–91. doi: 10.1002/ece3.6571 (PMC7487252; doi:10.1002/ece3.6571)
Supplement: Supplementary file 1 — Supinfo [file ECE3-10-9178-s001.pdf]

Supplementary Table: Summary of all specimens used for training and testing of the DL model with plant order, family, genus, species, climate region of the species, unique barcode identifier of the herbarium specimen, type of dataset the specimens were used for (TS=training set, UBST=unseen beyond the scope of training, UWST=unseen within the scope of training, VS=validation set), number of images taken from each specimen and average precision, recall and F-score values. The specimens in the table can be visualized in the virtual herbarium of Meise Botanic Garden following this link: http://www.botanicalcollections.be/specimen/*.

**Order**	**Family**	**Genus**	**Species**	**Climate region**	**Barcode***	**Data set**	**# images**	**Precision**	**Recall**	**F-score**
Ericales	Lecythidaceae	*Petersianthus*	*macrocarpus*	Tropical rainforest	BR0000013013363	TS	15	0.94	0.95	0.95
Ericales	Lecythidaceae	*Petersianthus*	*macrocarpus*	Tropical rainforest	BR0000013986889	TS	15	0.94	0.86	0.90
Fabales	Caesalpiniaceae	*Erythrophleum*	*suaveolens*	Tropical rainforest	BR0000013648268	TS	15	1.00	0.92	0.96
Fabales	Caesalpiniaceae	*Gilbertiodendron*	*dewevrei*	Tropical rainforest	BR0000013007010	TS	1	0.64	1.00	0.78
Fabales	Caesalpiniaceae	*Prioria*	*balsamifera*	Tropical rainforest	BR0000013003845	TS	14	0.81	0.94	0.87
Fabales	Caesalpiniaceae	*Prioria*	*balsamifera*	Tropical rainforest	BR0000013008680	TS	15	0.87	0.96	0.91
Fabales	Caesalpiniaceae	*Prioria*	*balsamifera*	Tropical rainforest	BR0000013277451	TS	15	0.89	0.95	0.92
Fabales	Caesalpiniaceae	*Prioria*	*oxyphylla*	Tropical rainforest	BR0000013007119	TS	15	0.97	0.93	0.95
Fabales	Caesalpiniaceae	*Prioria*	*oxyphylla*	Tropical rainforest	BR0000013989705	TS	15	0.98	0.95	0.97
Fabales	Mimosaceae	*Pentaclethra*	*macrophylla*	Tropical rainforest	BR0000013008734	TS	10	0.96	0.93	0.94
Fabales	Mimosaceae	*Pentaclethra*	*macrophylla*	Tropical rainforest	BR0000013013349	TS	26	0.72	0.69	0.70
Magnoliales	Annonaceae	*Polyalthia*	*suaveolens*	Tropical rainforest	BR0000013003586	TS	14	0.59	0.89	0.71
Malpighiales	Euphorbiaceae	*Ricinodendron*	*heudelotii*	Tropical rainforest	BR0000013004187	TS	15	0.89	0.95	0.92
Malpighiales	Guttiferae	*Garcinia*	*punctata*	Tropical rainforest	BR0000013002718	TS	12	0.92	0.81	0.86
Malpighiales	Guttiferae	*Garcinia*	*punctata*	Tropical rainforest	BR0000013013448	TS	11	0.68	0.98	0.80
Malpighiales	Guttiferae	*Mammea*	*africana*	Tropical rainforest	BR0000013008864	TS	8	0.96	0.95	0.95
Malpighiales	Irvingiaceae	*Irvingia*	*grandifolia*	Tropical rainforest	BR0000013008871	TS	3	0.52	0.77	0.62
Malvales	Sterculiaceae	*Cola*	*griseiflora*	Tropical rainforest	BR0000013002824	TS	12	0.92	0.93	0.92
Malvales	Sterculiaceae	*Cola*	*griseiflora*	Tropical rainforest	BR0000013010591	TS	29	0.96	0.92	0.94
Rosales	Moraceae	*Trilepisium*	*madagascariense*	Tropical rainforest	BR0000013005047	TS	15	0.86	0.99	0.92
Rosales	Ulmaceae	*Celtis*	*mildbraedii*	Tropical rainforest	BR0000013013431	TS	14	0.95	0.90	0.92

Santalales	Olacaceae	*Strombosiopsis*	*tetrandra*	Tropical rainforest	BR0000013008437	TS	12	0.75	0.92	0.82
Santalales	Olacaceae	*Strombosiopsis*	*tetrandra*	Tropical rainforest	BR0000013985943	TS	15	0.89	0.94	0.92
Sapindales	Meliaceae	*Carapa*	*procera*	Tropical rainforest	BR0000013004071	TS	11	0.87	0.96	0.91
Sapindales	Meliaceae	*Entandrophragma*	*candollei*	Tropical rainforest	BR0000013009335	TS	15	0.98	0.94	0.96
Sapindales	Meliaceae	*Entandrophragma*	*utile*	Tropical rainforest	BR0000013267070	TS	15	0.98	0.97	0.98
Sapindales	Meliaceae	*Entandrophragma*	*utile*	Tropical rainforest	BR0000013010959	TS	8	0.99	0.99	0.99
Sapindales	Meliaceae	*Entandrophragma*	*utile*	Tropical rainforest	BR0000013010966	TS	15	0.97	0.96	0.96
Sapindales	Meliaceae	*Entandrophragma*	*utile*	Tropical rainforest	BR0000013013615	TS	15	0.99	0.98	0.98
Sapindales	Meliaceae	*Trichilia*	*gigliana*	Tropical rainforest	BR0000013004613	TS	15	0.91	0.99	0.95
Sapindales	Meliaceae	*Trichilia*	*gigliana*	Tropical rainforest	BR0000013006976	TS	12	0.83	0.97	0.90
Sapindales	Meliaceae	*Trichilia*	*gigliana*	Tropical rainforest	BR0000013266608	TS	9	0.84	0.97	0.90
Asparagales	Asparagaceae	*Chlorophytum*	*ruahense*	Tropical moist deciduous forest	BR0000013289737	UBST	13	0.44	0.66	0.49
Asparagales	Asparagaceae	*Chlorophytum*	*ruahense*	Tropical moist deciduous forest	BR0000013289775	UBST	4	0.71	0.72	0.69
Asparagales	Asparagaceae	*Chlorophytum*	*orchidastrum*	Tropical rainforest	BR0000021758102	UBST	12	0.41	0.72	0.48
Asparagales	Asparagaceae	*Chlorophytum*	*orchidastrum*	Tropical rainforest	BR0000005718573	UBST	5	0.43	0.73	0.53
Asparagales	Asparagaceae	*Chlorophytum*	*orchidastrum*	Tropical rainforest	BR0000009026407	UBST	9	0.56	0.65	0.54
Asparagales	Orchidaceae	*Cyrtorchis*	*chailluana*	Tropical rainforest	BR000000989800	UBST	15	0.58	0.77	0.60
Asparagales	Orchidaceae	*Cyrtorchis*	*chailluana*	Tropical rainforest	BR000000989806	UBST	15	0.39	0.69	0.43
Asparagales	Orchidaceae	*Cyrtorchis*	*chailluana*	Tropical rainforest	BR000000989841	UBST	9	0.62	0.88	0.70
Ericales	Ebenaceae	*Diospyros*	*bipindensis*	Tropical rainforest	BR0000015396136	UBST	15	0.85	0.75	0.78
Ericales	Ebenaceae	*Diospyros*	*bipindensis*	Tropical rainforest	BR0000015396266	UBST	15	0.60	0.87	0.70
Ericales	Ebenaceae	*Diospyros*	*bipindensis*	Tropical rainforest	BR0000015396389	UBST	15	0.78	0.94	0.85
Ericales	Ebenaceae	*Diospyros*	*kirkii*	Tropical moist deciduous forest	BR0000014882197	UBST	15	0.68	0.79	0.71
Ericales	Ebenaceae	*Diospyros*	*kirkii*	Tropical moist deciduous forest	BR0000014882128	UBST	15	0.88	0.89	0.88
Ericales	Ebenaceae	*Diospyros*	*kirkii*	Tropical moist deciduous forest	BR0000014882166	UBST	15	0.52	0.80	0.61
Ericales	Ebenaceae	*Diospyros*	*mespiliformis*	Tropical shrubland/desert	BR0000014886386	UBST	15	0.88	0.94	0.91
Ericales	Ebenaceae	*Diospyros*	*mespiliformis*	Tropical shrubland/desert	BR0000014886744	UBST	15	0.79	0.93	0.85
Ericales	Ebenaceae	*Diospyros*	*mespiliformis*	Tropical shrubland/desert	BR0000014887031	UBST	15	0.79	0.94	0.85

Gentianales	Loganiaceae	*Strychnos*	*cocculoides*	Tropical moist deciduous forest	BR0000015876041	UBST	15	0.68	0.87	0.75
Gentianales	Loganiaceae	*Strychnos*	*cocculoides*	Tropical moist deciduous forest	BR0000015876096	UBST	15	0.77	0.94	0.84
Gentianales	Loganiaceae	*Strychnos*	*cocculoides*	Tropical moist deciduous forest	BR0000015875983	UBST	15	0.84	0.93	0.88
Gentianales	Loganiaceae	*Strychnos*	*spinosa*	Tropical shrubland/desert	BR0000013463687	UBST	15	0.74	0.96	0.83
Gentianales	Loganiaceae	*Strychnos*	*spinosa*	Tropical shrubland/desert	BR0000017158770	UBST	15	0.86	0.99	0.92
Gentianales	Loganiaceae	*Strychnos*	*spinosa*	Tropical shrubland/desert	BR000007000034	UBST	15	0.88	0.97	0.92
Gentianales	Loganiaceae	*Strychnos*	*usambarensis*	Tropical rainforest	BR0000013463878	UBST	15	0.79	0.74	0.74
Gentianales	Loganiaceae	*Strychnos*	*usambarensis*	Tropical rainforest	BR0000017162999	UBST	15	0.48	0.73	0.57
Gentianales	Loganiaceae	*Strychnos*	*usambarensis*	Tropical rainforest	BR0000017163002	UBST	15	0.85	0.77	0.79
Malpighiales	Euphorbiaceae	*Euphorbia*	*matabelensis*	Tropical moist deciduous forest	BR0000016000346	UBST	15	0.88	0.58	0.69
Malpighiales	Euphorbiaceae	*Euphorbia*	*matabelensis*	Tropical moist deciduous forest	BR0000016000377	UBST	4	0.76	0.29	0.38
Malpighiales	Euphorbiaceae	*Euphorbia*	*matabelensis*	Tropical moist deciduous forest	BR0000016000414	UBST	9	0.88	0.83	0.85
Poales	Poaceae	*Chloris*	*gayana*	Tropical moist deciduous forest	BR0000021566806	UBST	11	0.59	0.93	0.70
Poales	Poaceae	*Chloris*	*gayana*	Tropical moist deciduous forest	BR0000021566813	UBST	14	0.67	0.76	0.68
Poales	Poaceae	*Chloris*	*gayana*	Tropical moist deciduous forest	BR0000021566844	UBST	13	0.60	0.69	0.60
Poales	Poaceae	*Chloris*	*pilosa*	Tropical shrubland/desert	BR0000021568725	UBST	12	0.56	0.61	0.51
Poales	Poaceae	*Chloris*	*pilosa*	Tropical shrubland/desert	BR0000021568756	UBST	15	0.75	0.62	0.61
Poales	Poaceae	*Chloris*	*pilosa*	Tropical shrubland/desert	BR0000021568688	UBST	12	0.82	0.76	0.76
Poales	Poaceae	*Chloris*	*pychnotrix*	Tropical rainforest	BR0000024559362	UBST	15	0.67	0.42	0.48
Poales	Poaceae	*Chloris*	*pychnotrix*	Tropical rainforest	BR0000024559256	UBST	15	0.65	0.60	0.58
Poales	Poaceae	*Chloris*	*pychnotrix*	Tropical rainforest	BR0000024559348	UBST	9	0.89	0.30	0.43
Solanales	Convolvulaceae	*Ipomoea*	*cairica*	Tropical rainforest	BR0000016068896	UBST	14	0.43	0.93	0.55
Solanales	Convolvulaceae	*Ipomoea*	*cairica*	Tropical rainforest	BR0000016068902	UBST	9	0.60	0.97	0.72
Solanales	Convolvulaceae	*Ipomoea*	*cairica*	Tropical rainforest	BR0000016068971	UBST	8	0.55	0.79	0.64
Solanales	Convolvulaceae	*Ipomoea*	*eriocarpa*	Tropical moist deciduous forest	BR0000016788695	UBST	14	0.48	0.99	0.62
Solanales	Convolvulaceae	*Ipomoea*	*eriocarpa*	Tropical moist deciduous forest	BR0000016788718	UBST	12	0.64	0.97	0.76
Solanales	Convolvulaceae	*Ipomoea*	*eriocarpa*	Tropical moist deciduous forest	BR0000016788756	UBST	15	0.58	0.94	0.70
Solanales	Convolvulaceae	*Ipomoea*	*vagans*	Tropical shrubland/desert	BR0000017395212	UBST	10	0.64	1.00	0.78

Solanales	Convolvulaceae	*Ipomoea*	*vagans*	Tropical shrubland/desert	BR0000017395250	UBST	13	0.85	0.87	0.84
Solanales	Convolvulaceae	*Ipomoea*	*vagans*	Tropical shrubland/desert	BR0000017395281	UBST	4	0.65	0.99	0.79
Magnoliales	Annonaceae	*Polyalthia*	*suaveolens*	Tropical rainforest	BR0000013251994	UWST	13	0.96	0.99	0.97
Malpighiales	Euphorbiaceae	*Ricinodendron*	*heudelotii*	Tropical rainforest	BR0000013265229	UWST	10	0.94	0.97	0.95
Malpighiales	Guttiferae	*Mammea*	*africana*	Tropical rainforest	BR0000013627119	UWST	14	0.97	0.98	0.97
Malpighiales	Irvingiaceae	*Irvingia*	*grandifolia*	Tropical rainforest	BR0000013610258	UWST	2	0.83	0.51	0.63
Rosales	Ulmaceae	*Celtis*	*mildbraedii*	Tropical rainforest	BR0000013255886	UWST	14	0.89	0.86	0.87
Sapindales	Meliaceae	*Carapa*	*procera*	Tropical rainforest	BR0000013595463	UWST	3	0.95	0.98	0.96
Sapindales	Meliaceae	*Entandrophragma*	*candollei*	Tropical rainforest	BR0000013266899	UWST	14	0.99	0.95	0.97
Sapindales	Anacardiaceae	*Lannea*	*acida*	Tropical shrubland/desert	BR0000013714758	VS	15	0.95	0.88	0.91
Sapindales	Anacardiaceae	*Lannea*	*acida*	Tropical shrubland/desert	BR0000013848415	VS	15	0.89	0.93	0.91
Sapindales	Anacardiaceae	*Lannea*	*acida*	Tropical shrubland/desert	BR0000013848422	VS	15	0.97	0.88	0.92
Sapindales	Anacardiaceae	*Lannea*	*schweinfurthii*	Tropical moist deciduous forest	BR0000013719722	VS	15	0.99	0.92	0.95
Sapindales	Anacardiaceae	*Lannea*	*schweinfurthii*	Tropical moist deciduous forest	BR0000013719739	VS	15	0.73	0.93	0.81
Sapindales	Anacardiaceae	*Lannea*	*welwitschii*	Tropical rainforest	BR0000013721213	VS	15	0.89	0.88	0.88
Sapindales	Anacardiaceae	*Lannea*	*welwitschii*	Tropical rainforest	BR0000013721305	VS	15	0.92	0.92	0.91
Sapindales	Anacardiaceae	*Lannea*	*welwitschii*	Tropical rainforest	BR0000013721442	VS	15	0.85	0.91	0.88

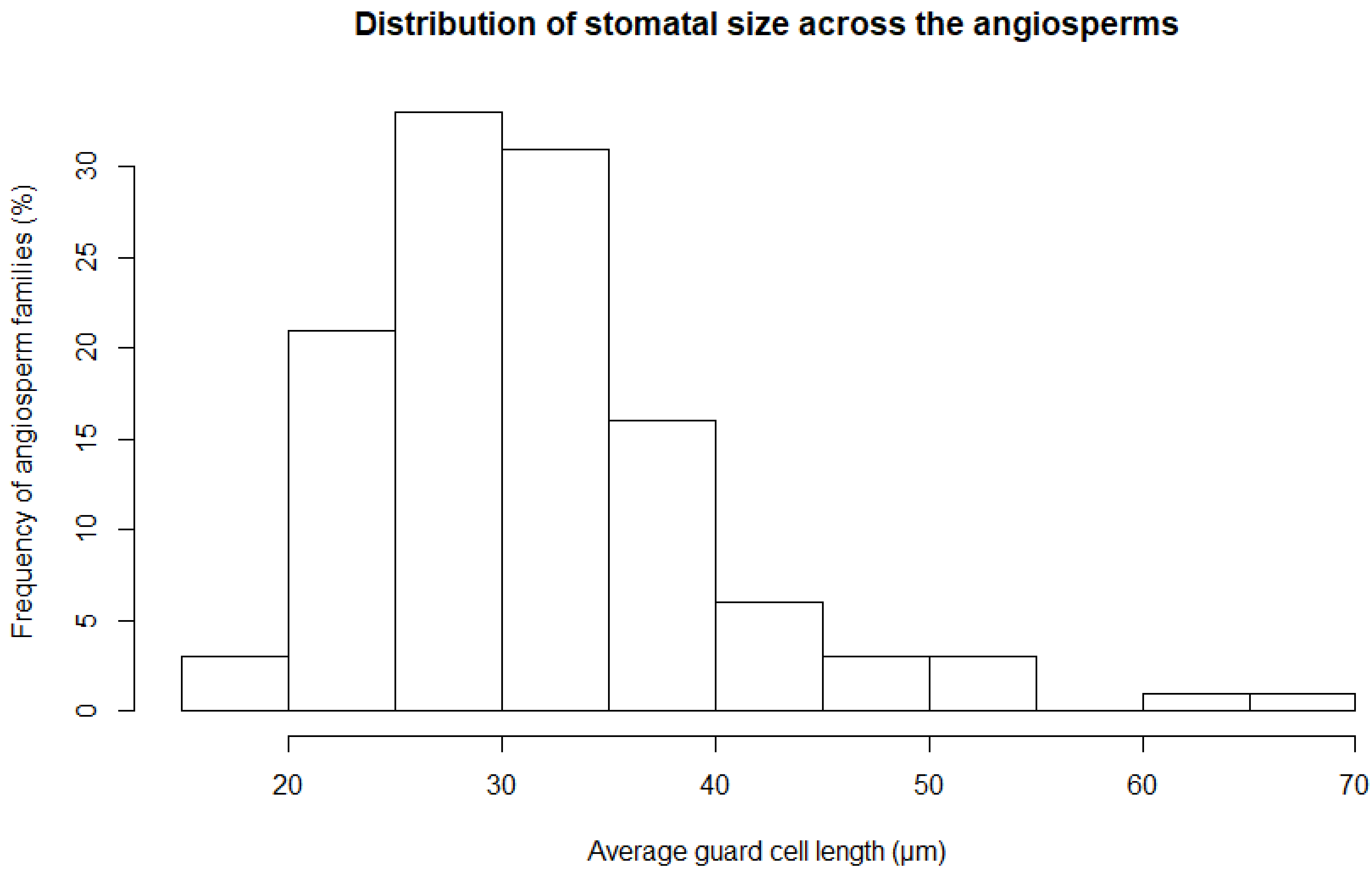

Supplementary Figure: Guard cell size distribution within angiosperms based on Hodgson et al. 2010 and Beaulieu et al. 2008.
